# Blueberry Extracts as a Novel Approach to Prevent Ozone-Induced Cutaneous Inflammasome Activation

**DOI:** 10.1155/2020/9571490

**Published:** 2020-08-13

**Authors:** Erika Pambianchi, Francesca Ferrara, Alessandra Pecorelli, Brittany Woodby, Mary Grace, Jean-Philippe Therrien, Mary Ann Lila, Giuseppe Valacchi

**Affiliations:** ^1^Plants for Human Health Institute, Animal Sciences Dept., NC Research Campus Kannapolis, NC State University, 28081, NC, USA; ^2^Department of Biomedical and Specialist Surgical Sciences, University of Ferrara, Ferrara, Italy; ^3^Plants for Human Health Institute, Food Bioprocessing & Nutrition Sciences Dept., NC Research Campus Kannapolis, NC State University, 28081, NC, USA; ^4^JP Therrien Consulting LLC, NC, USA; ^5^Department of Food and Nutrition, Kyung Hee University, Seoul, Republic of Korea

## Abstract

The World Health Organization estimates that 7 million people die every year due to pollution exposure. Among the different pollutants to which living organism are exposed, ozone (O_3_) represents one of the most toxic, because its location which is the skin is one of the direct tissues exposed to the outdoor environment. Chronic exposure to outdoor stressors can alter cutaneous redox state resulting in the activation of inflammatory pathways. Recently, a new player in the inflammation mechanism was discovered: the multiprotein complex NLRP1 inflammasome, which has been shown to be also expressed in the skin. The topical application of natural compounds has been studied for the last 40 years as a possible approach to prevent and eventually cure skin conditions. Recently, the possibility to use blueberry (BB) extract to prevent pollution-induced skin toxicity has been of great interest in the cosmeceutical industry. In the present study, we analyzed the cutaneous protective effect of BB extract in several skin models (2D, 3D, and human skin explants). Specifically, we observed that in the different skin models used, BB extracts were able to enhance keratinocyte wound closure and normalize proliferation and migration responses previously altered by O_3_. In addition, pretreatment with BB extracts was able to prevent ozone-induced ROS production and inflammasome activation measured as NRLP1-ASC scaffold formation and also prevent the transcripts of key inflammasome players such as CASP1 and IL-18, suggesting that this approach as a possible new technology to prevent cutaneous pollution damage. Our data support the hypothesis that BB extracts can effectively reduce skin inflammation and be a possible new technology against cutaneous pollution-induced damage.

## 1. Introduction

The last estimates of the World Health Organization (WHO) state that 9 out of 10 people living in urban areas are exposed to pollution levels above the healthy recommendations, leading to around 7 million deaths per year [1].

Pollution is a term used to describe a wide array of pollutants (ozone, diesel fuel exhaust, cigarette smoke, and heavy metals) to which living organisms are exposed, and among them, tropospheric ozone (O_3_) is one of the most toxic [2]. O_3_ concentrations can vary depending on altitude, seasonality, and the geographical location of the area (rural or urban); in some of the most polluted cities, O_3_ concentration can reach concentration between 0.5 ppm and 0.8 ppm [2–4].

O_3_ is a secondary pollutant because its formation is due to the interaction between the hydrocarbons and oxides of nitrogen released from car exhaust and sunlight (UV), leading to photochemical smog [2]. The effects of O_3_ exposure on target organs such as the respiratory tract have been investigated over the last 3 decades, and a strong correlation was clearly revealed between the development of respiratory conditions and ozone exposure. On the other hand, the effects that O_3_ has on the skin have been studied only recently [5–8] suggesting a link between the development of skin conditions and ozone exposure [2, 9–11]. Although O_3_ is not a radical per se, it is very reactive and its ability to induce tissue damage is mainly associated with its interaction with the cutaneous lipids present in the stratum corneum (SC), generating molecules such as hydrogen peroxide (H_2_O_2_) and lipid peroxidation products (4 hydroxynonenal (4HNE)) that can trigger an inflammatory response [12–16].

Recently, a new protein complex, the inflammasome, has been shown to be involved in several inflammatory tissue responses. It can be activated by a wide array of stimuli, such as pathogen and danger-associated molecular patterns (PAMPs and DAMPs), ionic flux, lysosomal damage, mitochondrial dysfunction, and the production of reactive oxygen species (ROS) [17].

Different inflammasomes have been so far identified (NLRP1, NLRP3, NLRC4, and AIM2); they are all multimeric protein complex part of the innate immune system and which rely on pattern recognition receptors (PPRs) to sense extracellular stimuli and a variety of stress factors [17, 18]. In particular, stimulation of inflammasomes NLRP1 and NLRP3 leads to the assembly of the components of this receptor (NOD-like sensor receptor (NLRs), apoptosis-associated speck-like adaptor protein (ASC), and procaspase 1) yielding to caspase 1-mediated activation which induces the secretion of proinflammatory cytokines such as IL-18 and IL-1*β* [17]. Chronic activation of the inflammasome has been correlated with the development of different conditions: atherosclerosis, autoinflammatory disease, Parkinson's and Alzheimer's disease [19–24], and recently also with skin diseases such as vitiligo, atopic dermatitis, acne, melanoma, pigmentation, and psoriasis [25–27].

Previous studies provided evidences that the NLRP1 (rather than the NLRP3) inflammasome is involved in epidermis inflammation [28, 29]. Canonical activation of the inflammasome requires the oligomerization of 3 different proteins: sensor receptor NLRP1, the speck-like receptor ASC, and procaspase 1. Once these interactions are established, the autocatalysis of the procaspase 1 in active caspase 1 induces the cleavage of the inactive zymogens pro-IL-18 and pro-IL-1*β* into their active forms [17]. Our previous work unveiled the ability of ozone (ranging from 0.4 to 0.5 ppm) to activate cutaneous NLRP1 inflammasome [30].

Cells are able to quench, to a certain extent, the production of ROS via endogenous antioxidant enzymes (glutathione peroxidase, catalase, and superoxide dismutase, including the thioredoxin and peroxiredoxin systems) [31–33]; nevertheless, this defensive system can be insufficient when the exposure to oxidative stimuli is particularly intense or long lasting, as can be the case when living in polluted environments [31, 34].

The use of topical interventions to improve skin defense against the outdoor environment has been studied for many years [15, 16, 35, 36]. Interventional studies indicate that it is possible to delay extrinsic skin aging and cutaneous damage and to improve skin conditions through the administration of specific natural compounds [37, 38] such as vitamin C and vitamin E (tocopherols) [14–16, 39–41], but more research needs to be done in providing more efficient compounds or even possibly mixture combining several natural extracts to be used against pollution-induced skin damage.

In the last few years, research on health benefits associated with blueberry (BB) dietary intake has risen sharply, due to evidence supporting blueberry's beneficial properties in reducing the risk of cardiovascular disease and type 2 diabetes, improving weight maintenance, and neuroprotection and most of all due to their significant antioxidant and anti-inflammatory properties thanks to their abundant polyphenolic compounds [42–44].

Blueberries are rich in polyphenolic compounds, which are widely distributed in nature and are important for plant survival since they attract pollinators and protect the fruit itself against various abiotic and biotic stress sources [45]. The main phytochemicals present in blueberry fruit are polyphenol compounds, including anthocyanins, proanthocyanidin, flavonols, and phenolic acids. Anthocyanins represent the major group of polyphenols in BB, including monoglycosides of delphinidin, cyanidin, petunidin, peonidin, and malvidin [46]. For cultivated blueberries, anthocyanins are mostly concentrated in the blueberry skin, giving the characteristic indigo color; therefore, a small-sized blueberry would have a relatively higher skin surface (with respect to the berry volume) and consequently higher anthocyanin content compared to another bigger blueberry [47]. Wild lowbush blueberry (*Vaccinium angustifolium* Aiton), typically smaller than most cultivated blueberries, contains anthocyanins in both the skin and the flesh of the berry fruit [48, 49].

The topical application of various blueberries has been studied to reduce telangiectasias, wrinkle formation, and skin aging [50–52], and its topical and medicinal use has been recorded in the Traditional Ecological Knowledge of Native American pharmacopoeia [53]; but the potential advantageous mechanisms of topical application against pollution-induced damage have never been evaluated, especially for *Vaccinium* species.

Since ozone exposure has already been associated with the development and exacerbation of inflammatory skin diseases ([2, 54, 55]), we hypothesized that the use of blueberry (BB) extracts would be able to quench the ozone-induced activation of cutaneous inflammasome through the prevention of redox imbalance.

In the present study, we have observed that BB extract was able to prevent ozone-induced inflammasome-related genes and proteins levels and also the oligomerization of the inflammasome components. In addition, BB extract pretreatment was efficient in improving epithelial wound healing and decreasing oxidative markers related to ozone exposure.

## 2. Materials and Methods

### 2.1. Ozone Generator

O_3_ was generated via electrical corona arc discharge from O_2_ and combined with ambient air to flow into a plexiglass box (ECO3 model CUV-01, Italy, Model 306 Ozone Calibration Source, 2B Technologies, Ozone Solution), as previously described [56] and constantly monitored by an ozone detector. The dose was based on our previous studies [15].

### 2.2. Plant Material

A uniform composite of ripe wild lowbush blueberries (*V. angustifolium*, Aiton) grown in maritime provinces of Canada (Quebec, Nova Scotia, Prince Edward Island, and New Brunswick) and the State of Maine (USA) was provided by the Wild Blueberry Association of North America (Old Town, ME, USA). Blueberries (BB) were individually quick-frozen (IQF) within hours of harvest and then stored at −70°C until they were shipped to North Carolina on dry ice, where they were then stored at -80°C. Frozen BB samples were lyophilized before extraction.

### 2.3. Extraction

Extraction of lyophilized BB was conducted according to our published procedures with minor modifications [57]. Ground freeze-dried BB (5 g) were placed in 50 ml centrifuge tubes embedded in ice and homogenized in 30 ml of cold extraction solvent (70% aqueous methanol, 0.5% acetic acid) using a Pro 250 homogenizer (Pro Scientific Inc. Oxford, CT, USA) for 4 min. The obtained slurry was centrifuged (Sorvall RC-6 Plus, Asheville, NC, USA) for 10 min at 3452 × g force at 4°C. The supernatant was collected in a 100 ml volumetric flask. The residue was then extracted twice with same solvent, and supernatants were collected all together and brought to a final volume of 100 ml with the extraction solvent. An aliquot was evaporated to an aqueous solution and lyophilized [58].

### 2.4. Blueberry Extract Preparation

BB frozen extract powder was solubilized in a volume of dimethylsulfoxyde (DMSO) (Thermo Fisher Scientific, USA, cat no. 20688 99.5%) needed to reach a primary concentration of 100 mg/ml (stock), aliquoted, and stored at -80°C. For each experiment, a new freshly made BB treatment was prepared in complete media from the stock, via serial dilutions: ranging from 0.1 *μ*g/ml to 100 *μ*g/ml based on the skin model utilized. The specific volumes utilized to prepare the serial dilutions of BB extract were also utilized to make the negative controls of DMSO (final concentration 0.01%) in complete media.

### 2.5. Keratinocytes Culture, BB Pretreatment, and Ozone Exposure

HaCaT cells (AddexBio, USA) were cultured in high-glucose (4.5%) Dulbecco's modified Eagle's medium (Corning, USA) supplemented with 10% FBS (Sigma, USA), 100 U/ml penicillin, and 100 *μ*g/ml streptomycin (Gibco, USA) (complete media) and grown at 37°C in 5% CO_2_ and 95% air. For LDH, BrdU, and DCFDH, 10,000 cells were seeded in 96-well plate dishes (Corning, USA); 250,000 cells were seeded into 12-well plates (Corning, USA) to perform the scratch-wound assay; and 1 million cells were instead seeded in 6 cm^2^ petri dishes (Corning, USA) for RT-PCRs. After 8 hours from the seeding, HaCaT were starved with high-glucose Dulbecco's modifies Eagle's medium supplemented with 1% FBS (Sigma, USA), 100 U/ml penicillin, and 100 *μ*g/ml streptomycin (Gibco, USA). On the second day, in the morning, the media were removed and either control medium containing the preestablished volume of DMSO (0.01%) or BB extracts were added at a dose of 10 *μ*g/ml and left for 24 h while cells were incubated at 37°C in 5% CO_2_ and 95% air. In the third morning, the BB pretreatment media and the control media were discarded and complete media were added; then, cells were placed in a plexiglass box connected to the ozone generator and exposed to ozone for 1 h at the dose of 0.5 ppm. After the ozone exposure, samples were harvested for the biochemical and immunochemical assays.

### 2.6. 3D Skin Model Treatment and Ozone Exposure

3D skin model “EpiDerm” (reconstructed human epidermis (RHE)) was purchased from MatTek corporation (EpiDerm, EPI-200). Upon arrival, the 24 inserts containing 3D skin tissues were transferred into 6-well plates prefilled with 1 ml of MatTek Assay medium (provided by MatTek corporation, USA), according to the manufacturer's instructions as previously described [59]. The plates were placed in the incubator overnight (5% CO_2_, 37°C) for recovery. On the day after, either control medium containing the preestablished volume of DMSO or BB extracts were added at the dose 100 *μ*g/ml and left for 24 h. On day 3, the media were discarded and complete media were added to the tissues and exposed to O_3_ for 5 h at the dose 0.5 ppm. Protein and RNA were collected right after exposure (T0), 6 h (T6), and 24 h (T24) postexposure.

### 2.7. Human Skin Explant Treatment and Ozone Exposure

Healthy human skin was purchased from Hunstad/Kortesis/Bharti Cosmetic Surgery clinic. 12 mm punch biopsies were taken from the skin and excised using sterile scissors; subcutaneous tissue was removed with a scalpel, and the biopsies were rinsed with Phosphate-Buffered Saline (PBS) containing antibiotics/antimycotic using a sterile technique [30]. Then, the skin explants were transferred to 6-well plates and cultured in complete media, at 37°C in 5% CO_2_ and 95% air. The morning after, either control medium containing the preestablished volume of DMSO or BB extracts were added at the dose of 100 *μ*g/ml and incubated for 24 h. On day 3, the tissues were placed in a plexiglass box connected to the ozone generator and exposed for 5 h at the dose of 0.5 ppm. Skin samples were collected, dehydrated, and embedded in paraffin to perform immunohistochemistry at 0 h, 6 h, and 24 h upon ozone exposure.

### 2.8. Lactate Dehydrogenase (LDH) Cytotoxicity Assay

After 24 h of BB pretreatment at the doses of 0.1, 0.5, 1, 5, and 10 *μ*g/ml for HaCaT and 10, 50, and 100 *μ*g/ml for RHE, supernatants from both the 2D and 3D models were collected, and cytotoxicity was calculated by measuring the amount of the enzyme lactate dehydrogenase (LDH) released in the cytosol, according to manufacturer's instructions (Roche, USA, cat no. 11644793001). Levels of LDH released into the media from keratinocytes and RHE were normalized to the positive control Triton X100, considered 100% LDH release [60].

### 2.9. Scratch Wound Healing Assay

After seeding (250,000 HaCaT cells per 12-well plate), BB pretreatment, and ozone exposure, the adherent monolayer of human keratinocytes was mechanically scratched with a sterile p200 pipette tip and cellular debris were washed off with PBS (Corning, USA). Pictures of the wound area were taken at different time points (0 h, 18 h, and 36 h upon ozone exposure) in three different places, using AxioVision software (40x magnification). Quantification of the wound width was determined by analysis with ImageJ software (National Institutes of Health, Bethesda, MD, USA) and compared to the wound area at 0 h, arbitrarily set at 100 [61, 62].

### 2.10. In Vitro Cell Migration Assay

HaCaT cells were seeded in 10 cm^2^ petri dishes and pretreated with BB at the dose 10 *μ*g/ml for 24 h; then, the cells were detached and 50,000 cells were resuspended in serum-free media and seeded in 8 *μ*m pore size transwells (QCMTM 24-well Colorimetric Cell Migration Assay kit, Millipore, USA) coated with 0.15 mg/ml bovine collagen IV. After 30 min, 650 *μ*l of complete media was added at the bottom of each well, acting as a chemoattractant. Following ozone exposure (0.5 ppm for 1 h) the transwell inserts were fixed for 10 min with 70% ethanol, stained with 0.02% of Coomassie Blue for 15 min, and rinsed with double-distilled water. HaCaT cells left unmigrated in the upper part of the transwell were gently removed with a cotton swab, and pictures of 3 randomly selected fields were captured using AxioVision software (20x magnification). Automated quantification of the migrated cells was performed using ImageJ program as follows: conversion to grayscale of the image, removal of noise, and adjustment of brightness and contrast (min = 87, max = 167); then, a Phansalkar threshold and watershed were applied [62].

### 2.11. In Vitro Cell Proliferation Assay

Cellular proliferation in HaCaT cells was evaluated by bromodeoxyuridine (BrdU) incorporation assay (Roche, USA, cat no. 11647229001). After seeding (10,000 cells/well in 96-well plate) and upon BB pretreatment and ozone exposure, 20 *μ*l of BrdU labeling solution/well was added to the cells and incubated for 24 h (5% CO_2_, 37°C); following, the cells were dried and fixed and the cellular DNA was denatured allowing a better detection by the antibody of the already incorporated BrdU [63]. Then, as indicated by the manufacturer, the monoclonal anti-BrdU peroxidase-conjugated antibody was added to each well and incubated at room temperature for 90 min. The cells were then rinsed with PBS, and the bound peroxidase was photometrically detected after 30 min via substrate reaction and quantified by measuring the absorbance produced at 370 nm (reference wavelength 492 nm).

### 2.12. Dichlorofluorescein (DCF) Assay

10,000 cells were seeded in a 96-well plate, starved overnight, and pretreated with BB 10 *μ*g/ml for 24 h. Prior to ozone exposure, the BB pretreatment was removed and cells were washed with warm PBS. Then, 2′,7′ acetylated dichlorofluorescein (DCF) (Invitrogen, Thermo Fisher Scientific, USA, cat no. C2938) was resuspended in PBS to reach the concentration 10 *μ*M and incubated with the cells, in the dark, for 30 min at 37°C, to allow the internalization of the fluorescent probe in the cells. Following, the DCF was removed and 100 *μ*l/well of Dulbecco's modified Eagle's medium without Red Phenol (Corning) supplemented with 1% of FBS (Sigma), 100 U/ml penicillin, and 100 *μ*g/ml streptomycin (Gibco) was added. The cells were then exposed to ozone (1 h at 0.5 ppm), and the fluorescence of oxidized DCF dye was evaluated 1 hour after ozone exposure as previously described [64].

### 2.13. ASC Oligomerization Assay

HaCaT cells (1 × 10^6^) were grown in 6 cm^2^ petri dishes, starved overnight, and pretreated with BB 10 *μ*g/ml for 24 h. Just prior to ozone exposure, the BB pretreatment was removed, and cells were exposed to ozone for 1 h at 0.5 ppm and collected after 0 h, 1 h, and 3 h. Cells were washed in cold PBS, gently detached with a scraper, and centrifuged for 5 min at 1500 × g. The cell pellet was resuspended in 500 *μ*l of cold lysis buffer (containing Hepes, KOH 20 mM (pH 7.5), KCl 150 mM, NP-40 1%, 1% protease inhibitor cocktails (Sigma), and PMSF 0.1 mM). Following, cell lysates were centrifuged at 1800 × g at 4°C for 8 min, and 30 *μ*l of the lysates was collected as input for Western blot analysis (later resuspended in 2X Laemmli buffer, 20% beta-mercaptoethanol), while the remaining volume was centrifuged again for 10 min at 5000 × g at 4°C. Upon centrifugation, to induce crosslinking of the oligomers, the lysates were resuspended in 500 *μ*l of cold PBS containing disuccinimidyl suberate (DSS) (Thermo Fisher Scientific, USA, CAS 68528-80-3 Alfa Aesar) and incubated at RT for 30 min on a rotator. Following, the cellular samples were centrifuged for 10 min at 2500 rpm at 4°C, and the crosslinked pellets were then resuspended in 1X Laemmli buffer and 10% beta-mercaptoethanol. The input and crosslinked samples were boiled for 10 min at 95°C and then analyzed by running samples on a 4–12% SDS-PAGE gel. Bands were digitized, and densitometric analysis was performed using ImageJ software.

### 2.14. Immunocytochemistry

HaCaT cells were grown on coverslips (10,000 cells), starved overnight, and pretreated with BB 10 *μ*g/ml for 24 h. Right before ozone exposure, the BB pretreatment was removed, and cells were exposed to ozone for 1 h, at 0.5 ppm, collected at the different time points, and fixed in 4% paraformaldehyde (PFA) in PBS for 30 min at 4°C. Permeabilization was performed with 0.25% Triton X100 in PBS and then blocked in PBS-BSA (Bovine Serum Albumin, Sigma) 1% at room temperature for 1 h. ASC and NLRP1 primary antibodies were then incubated overnight (ASC, cat NBP1-78977 NovusBio, USA 1 : 100 in 0.25% BSA/PBS and NLRP1 sc-166368 Santa Cruz, USA 1 : 50 in 0.25% BSA/PBS) at 4°C. The following day, the coverslips were incubated with the fluorochrome-conjugated secondary antibodies (A11004 Alexa Fluor 568, A11008 Alexa Fluor 488) for 1 h at room temperature. DAPI (D1306 Invitrogen, USA) was utilized to stain the nuclei (1 min at room temperature). Then, coverslips were mounted onto glass slides using PermaFluor ˙Aqueous Mounting Medium (TA-006-FM Thermo Fisher Scientific) and examined using a Zeiss Z1 AxioObserver LSM10 confocal microscope equipped at 40x magnification. ASC specks were analyzed via ImageJ, and ASC speck number was correlated with the number of nuclei present in the correspondent picture.

As for the skin explants, 4 *μ*m sections were punched and deparaffinized with the use of xylene and rehydrated in decreasing alcohol gradients. 10 mM sodium citrate buffer (AP-9003-500, Thermo Fisher Scientific) (pH 6.0) was utilized at a subboiling temperature (microwave settings 500 W, 10 min) to induce antigen retrieval and then cooled off for 20 min. Following, 2 washes × 5 min with PBS were performed and then sections were blocked with 5% BSA in PBS at room temperature for 45 min, then incubated overnight at 4°C with primary antibodies for ASC (cat NBP1-78977 NovusBio, USA) dil. 1 : 100 in PBS with 2% BSA, NLRP1 (sc-166368 Santa Cruz, USA) at 1 : 50 dilution in 2% BSA in PBS, and 4HNE (AB5605 Millipore Corp., USA) at 1 : 400 dilution in 2% BSA in PBS. The following day, 3 washes with PBS of 5 min each were performed, followed by the incubation in the dark with fluorochrome-conjugated secondary antibodies (A11004 Alexa Fluor 568, A11008 Alexa Fluor 488 and A11055 Alexa Fluor 488) at 1 : 500 dilutions in PBS with 2% BSA for 1 h at room temperature. After 3 washes (5 min each) with PBS, the sections were mounted onto glass slides using PermaFluor mounting media (Thermo Fisher Scientific) and images were collected by a Zeiss LSM10 microscope equipped with 40x magnification.

### 2.15. Protein Extraction

Cell lysates were extracted in ice-cold lysis buffer containing 50 mM Tris (pH 7.5), 150 mM NaCl, 10% glycerol, 1% Nonidet P-40, 1 mM EDTA, 0.1% SDS, 5 mM nethylmaleamide (Sigma), and protease and phosphatase inhibitor cocktails (Sigma). Lysates were then centrifuged for 15 min at 4°C and 12700 rpm, supernatants were collected, and soluble protein concentration was measured via Quick Start Bradford protein method (Bio-Rad, USA). RHE were collected and harvested in Tissue Protein Extraction Reagent (T-PERTM) (Thermo Fisher Scientific) supplemented with 1% of protease and phosphatase inhibitor cocktails (Sigma, USA). Three cycles of freezing/thawing by moving from liquid nitrogen to 37°C were performed, and following, centrifugation at 12700 rpm for 15 min at 40°C was assessed. Protein content was evaluated on the RHE lysates via Bradford assay (Bio-Rad).

### 2.16. Western Blot Assay

4–12% polyacrylamide SDS gels were loaded with equivalent amounts of protein (previously denatured for 10 min at 95°C), which were then separated by molecular size. The gel was electroblotted onto nitrocellulose membranes, and blocking was performed with Tris-buffered saline, pH 7.5, containing 0.5% Tween 20 and 5% nonfat milk, for 1 h at room temperature. After overnight incubation with the antibody caspase 1 (2225S cell signaling, USA) diluted 1 : 1000 in TBS-T with 1% nonfat milk (Bio-Rad, USA), the membranes were incubated for 1 h with the secondary antibody conjugated with horseradish peroxidase and the signal was detected by chemiluminescence (Bio-Rad, USA). Beta-actin (A3854 Sigma, USA) was used for loading control. Bands were digitalized, and densitometry analysis was evaluated via ImageJ software.

### 2.17. RNA Extraction and Quantitative Real-Time PCR (q-rtPCR)

For HaCaT cells and RHE, total RNA extraction was performed via the Aurum Total RNA Mini Kit with DNase digestion (Bio-Rad), according to the manufacturer's protocol. Specifically, for the RHE, 700 *μ*l of lysis buffer provided by the kit was added and the tissues were homogenized with Precellys tissue homogenizer (9 cycles of 30 s with a 30 s break at 8000 rpm at 4°C). The same kit was utilized to extract total RNA from HaCaT samples. cDNA was then generated from 1 *μ*g of total RNA, using the iScript cDNA Synthesis Kit (Bio-Rad). Evaluation of the mRNA levels of ASC, Caspase 1, and IL-18 genes was assessed via quantitative real-time PCR using SYBR® Green Master Mix (Bio-Rad) on a LightCycler® 480 Real-Time PCR System (Roche), according to the manufacturer's protocol. Gene expression was quantified via the number of cycles obtained to reach a predetermined threshold value in the intensity of the PCR signal (CT value). Beta-actin was employed as the reference gene, and the samples were compared using the relative cycle threshold (CT). After normalization, the fold change was determined using the 2^-*ΔΔ*CT^ method. The primers used are listed here: (*β*-actin forward ATTGCCGACAGGATGCAGA/reverse AGTACTTGCGCTCAGGAGGA, ASC forward ATGCGCTGGAGAACCTGA/reverse TCTCCAGGTAGAAGCTGACCA, Caspase 1 forward CCGTTCCATGGGTGAAGGTA/reverse TGCCCCTTTCGGAATAACGG, and IL-18 forward TGCAGTCTACACAGCTTCG/reverse ACTGGTTCAGCAGCCATCTT).

### 2.18. Statistical Analysis

Each of the variables tested is expressed as mean ± standard deviation (SD) of three independent experiments.

Statistical analysis was performed via GraphPad Prism 6 software (GraphPad Software Inc., La Jolla, CA, USA). Differences between groups were evaluated by analysis of variance (ANOVA) for single time point or by two-way ANOVA when different time points were included, followed by Tukey's post hoc test. A *p* value < 0.05 was considered statistically significant.

## 3. Results

### 3.1. Cytotoxic Evaluation of Blueberry (BB) Extract in 2D and 3D Cutaneous Models

**Figure 1:**
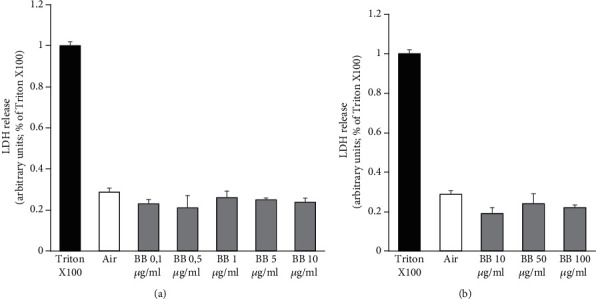
Cytotoxic evaluation of blueberry (BB) extract in 2D and 3D cutaneous models. (a) Keratinocytes cells were pretreated with different doses of BB (0.1, 0.5, 1, 5, and 10 *μ*g/ml) for 24 h. (b) 3D models were pretreated with BB doses ranging from 10 to 100 *μ*g/ml. Cytotoxicity was calculated into the supernatant of pretreated 2D and 3D models, by measuring the amount of LDH released from the cytosol. Data are the average ± SD of three independent experiments.

The first step of our study was the evaluation of the cytotoxicity of the BB extracts in our 2D and 3D cell culture models. Human keratinocytes and skin 3D models (RHE) were pretreated for 24 hours with different doses of BB extracts (0.1, 0.5, 1, 5, and 10 *μ*g/ml for the HaCaT and 10, 50, and 100 *μ*g/ml for the RHE), and cytosolic LDH released was evaluated in the supernatant. Our results showed that BB treatment did not affect cellular viability at all the doses tested in both the models. The average release of LDH for HaCaT cells was around 20% (Figure 1(a)) and 18% for the RHE (Figure 1(b)) with respect to the 100% cell death (Triton X100). Based on these results, we have decided to use the following doses of BB: 10 *μ*g/ml for HaCaT and 100 *μ*g/ml for RHE models.

### 3.2. Effect of Blueberry (BB) Extract on Ozone Modulation of Keratinocyte Migration, Proliferation, and H_2_O_2_ Production

In our previous study, we demonstrated that O_3_ and other pollutants are able to impair the skin repair abilities [65, 66]; therefore, next, we wanted to evaluate the potential properties of BB extract in improving the wound closure impairment induced by O_3_.

**Figure 2:**
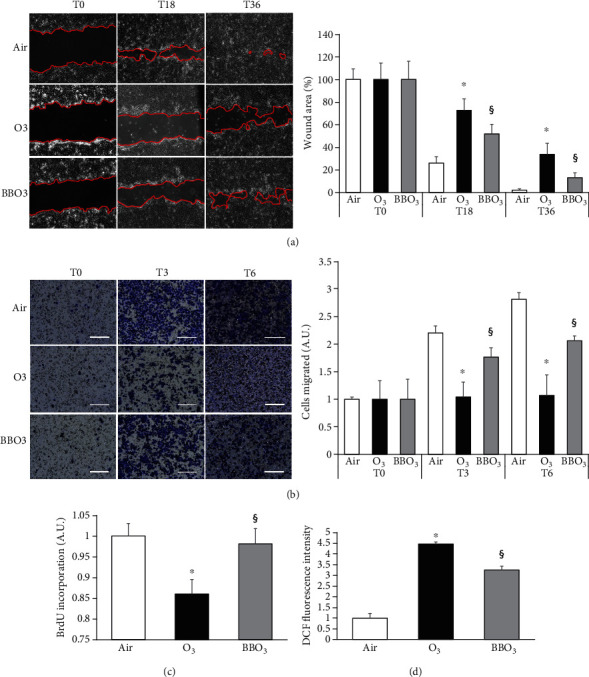
Effect of blueberry (BB) extract on ozone modulation of keratinocytes migration, proliferation, and ROS production. (a) Scratch was performed on confluent monolayer of HaCaT cells, and pictures were taken to measure wound area at different time points (0-18-36 h). On the left, depiction of the wound after 0, 18, and 36 h. On the right, quantification of the wound area at each time point via ImageJ. Data are shown as percent of 0 h. (b) Representative depiction of migration experiment performed on HaCaT cells, scale bar 200 *μ*m. Cells were seeded in 8 *μ*m pore size transwells, exposed to O_3_, and incubated for 0, 3, and 6 h. After fixation, migrated cells were stained with 0.02% Coomassie Blue. On the right, ImageJ quantification of the migrated cells after 0, 3, and 6 h from O_3_ exposure. Data are shown as average of 6 picture fields (20x magnification). (c) The growth response of HaCaT cells pretreated with BB was assessed after 24 h after O_3_ exposure by BrdU incorporation. (d) H_2_O_2_ production was evaluated via DCF after 1 h upon O_3_ exposure (1 h at 0.5 ppm) in HaCaT cells pretreated with BB. Data are the results of three independent experiments. ^∗^*p* < 0.05 air vs. O_3_, ^§^*p* < 0.05 O_3_ vs. BBO_3_ by one-way or two-way ANOVA.

As shown in Figure 2(a), exposure to O_3_ (0.5 ppm for 1 h) significantly impairs the ability of the keratinocytes to recover the scratch wound. Indeed, after ozone exposure, at 18 h time point, the wound was still 75% open with respect to the 30% of the control. This difference was still evident after 36 h of ozone exposure, where the wound was still 35% open while the control was completely recovered. Of notice, 24 h pretreatment with BB extracts significantly improved the keratinocyte wound closure ability with 50% and 15% open wound at 18 h and 36 h, respectively.

Because the scratch-wound assay is not able to discriminate between a proliferative and migratory effect, we decided to further evaluate both of these cellular responses in our experimental conditions.

As depicted in Figure 2(b), O_3_ exposure decreased by 50% the migratory property of the cells after 3 h of exposure and over 50% at 6 h time point. Also, in this case, BB extract pretreatment was able to completely abolish the effect of O_3_ exposure at 3 h time point and to improve the migratory efficiency of about 50% at 6 h.

Similar response was observed also for the proliferative assay as depicted in Figure 2(c). O_3_ exposure reduced HaCaT proliferation by 15%, and BB pretreatment was able to rescue this effect.

Considering the antioxidant properties of BB, we have tested whether BB were able to prevent O_3_-induced H_2_O_2_ formation. As shown in Figure 2(d), keratinocyte ROS production after 1 h post-O_3_ exposure was 5-fold higher compared to the control, and the pretreatment with BB extracts significantly suppressed this increase (circa 25%).

### 3.3. Blueberry (BB) Extract Prevents O_3_-Induced Activation of Inflammasome in 2D Skin Models

Cellular proliferative and migratory alterations are phenomena that are present in several inflammatory skin conditions [67, 68]. We have recently showed the ability of O_3_ to induce inflammasome activation and oligomerization [30]; now, we wanted to assess whether BB pretreatment could prevent this effect.

**Figure 3:**
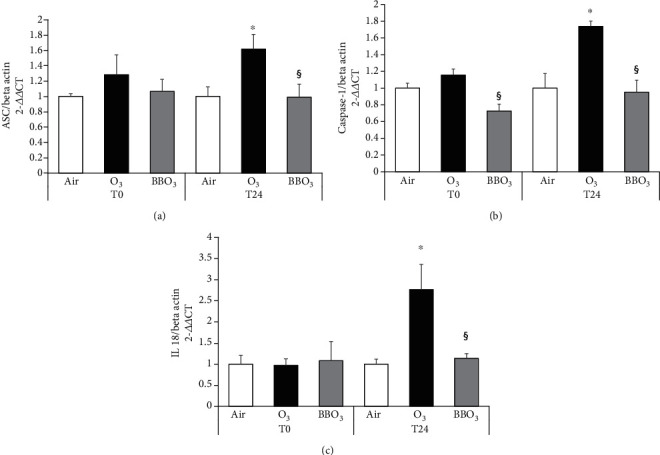
Blueberry (BB) extract prevents O_3_-induced activation of inflammasome in 2D skin models. HaCaT transcript levels for (a) ASC, (b) Caspase 1, and (c) IL-18 at 0 and 24 h. Data are the results of three independent experiments. ^∗^*p* < 0.05 air vs. O_3_, ^§^*p* < 0.05 O_3_ vs BBO_3_ by two-way ANOVA.

As showed in Figures 3, 24 h after O_3_ exposure, there was a significant increase in the transcript levels of key players in the inflammasome activation in HaCaT cells. As depicted in Figure 3(c), IL-18, which is an end product of the inflammasome activation, increased around 5-fold at 24 h time point; while Caspase 1 (Figure 3(b)) and ASC (Figure 3(a)) were already induced right after the O_3_ exposure (T0) and further increased at the later time point (T24). Of note, pretreatment with BB extracts was able to prevent the induction of ASC (Figure 3(a)), Caspase 1 (Figure 3(b)), and IL-18 (Figure 3(c)) in keratinocytes.

### 3.4. Blueberry (BB) Extract Prevents O_3_-Induced Activation of Inflammasome in 3D Skin Models

Since Caspase 1 is the cardinal player of the inflammasome, which actively cleaved the cytokine proforms, we evaluated its transcripts and protein levels also in the 3D model (RHE) [69].

**Figure 4:**
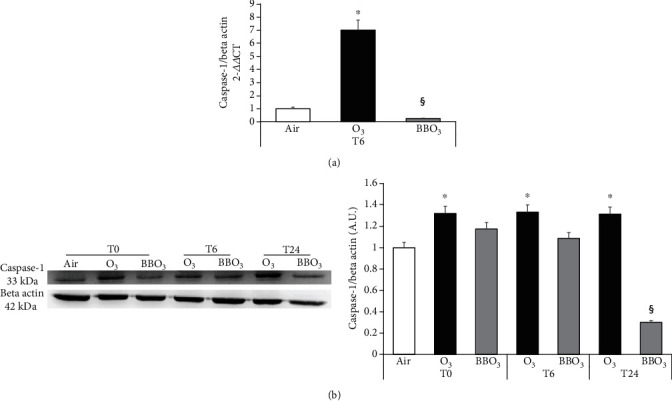
Blueberry (BB) extract prevents O_3_-induced activation of inflammasome in 3D skin models. RHE transcript levels for Caspase 1 (a). Protein levels of Caspase 1 in RHE at 0, 6, and 24 h after O_3_ exposure (b), on the right, relative quantification via ImageJ analysis. Data are the results of two independent experiments. ^∗^*p* < 0.05 air vs. O_3_, ^§^*p* < 0.05 O_3_ vs. BBO_3_ by two-way ANOVA.

As expected, the RHE confirmed the keratinocyte results. Indeed, O_3_ exposure clearly induced a remarkable increase in Caspase 1 transcripts and protein levels at the different time points analyzed (6 h for mRNA and 0 h, 6 h, and 24 h for proteins). Also, in this case, BB extract pretreatment clearly decreased the O_3_ effect, especially after 24 h, with a 4.5-fold decrease in caspase 1 protein levels (Figure 4(a) and 4(b)).

### 3.5. Blueberry (BB) Extract Prevents O_3_-Induced Inflammasome Oligomerization in HaCaT Cells

The activation of the NLRP1 inflammasome occurs only following the oligomerization of the scaffold-forming components. To evaluate the effect of BB extract on the oligomerization and inflammasome activation in keratinocytes upon O_3_ exposure, we performed immunofluorescence using different dyes (green for ASC, red for NLRP1).

**Figure 5:**
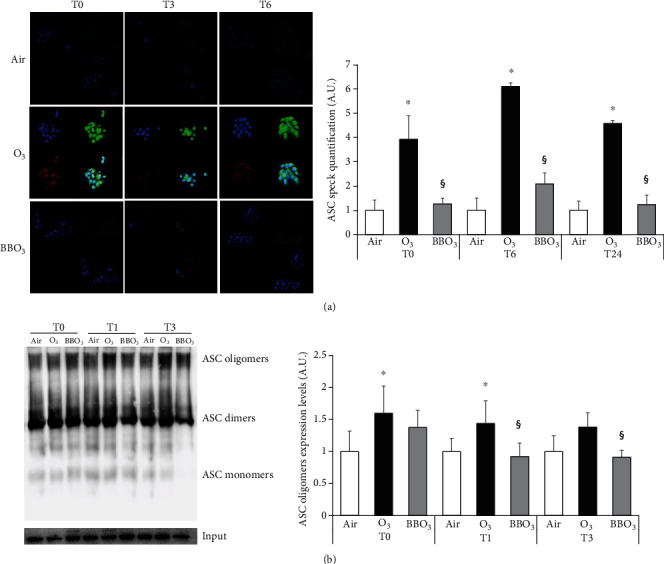
Blueberry (BB) extract prevents O_3_-induced inflammasome oligomerization in HaCaT cells. (a) Immunofluorescence staining of ASC (green), NLRP1 (red), DAPI (blue), and merge in HaCaT cells pretreated with 10 *μ*g/ml of BB for 24 h then exposed to 0.5 ppm of O_3_ for 1 h at 0, 3, and 6 h postexposure (40x magnification). On the right, ASC speck formation was quantified using ImageJ. (b) ASC oligomers, dimers, monomers, and input protein levels in keratinocytes at 0, 1, and 3 h after O_3_ exposure (1 h, 0.5 ppm). On the right, depiction of ASC oligomers and dimer quantification using ImageJ. Data are the results of three independent experiments. ^∗^*p* < 0.05 air vs. O_3_, ^§^*p* < 0.05 O_3_ vs. BBO_3_ by two-way ANOVA.

We observed increased perinuclear colocalization of ASC and NLRP1 upon O_3_ exposure at 0 h, 3 h, and 6 h, and the BB extract pretreatment significantly decreased the oligomerization of the scaffold (Figure 5(a)). Therefore, it is possible to hypothesize that BB extracts somehow are able to prevent the formation of ASC oligomers which is the first step for the inflammasome activation [70].

ASC oligomerization assay confirmed these results as shown in Figure 5, where it is possible to appreciate an increase in ASC oligomer and dimer formation (15%) after O_3_ exposure while this effect was almost completely rescued by BB extracts (Figure 5(b)).

### 3.6. Blueberry (BB) Extract Prevents O_3_-Induced Inflammasome Activation and Oxidative Stress in *Ex Vivo* Human Skin Biopsies

To further validate our previous data on 2D and 3D skin models, we decided to perform our experiments on a more complete cutaneous model represented by the *ex vivo* human skin explants.

**Figure 6:**
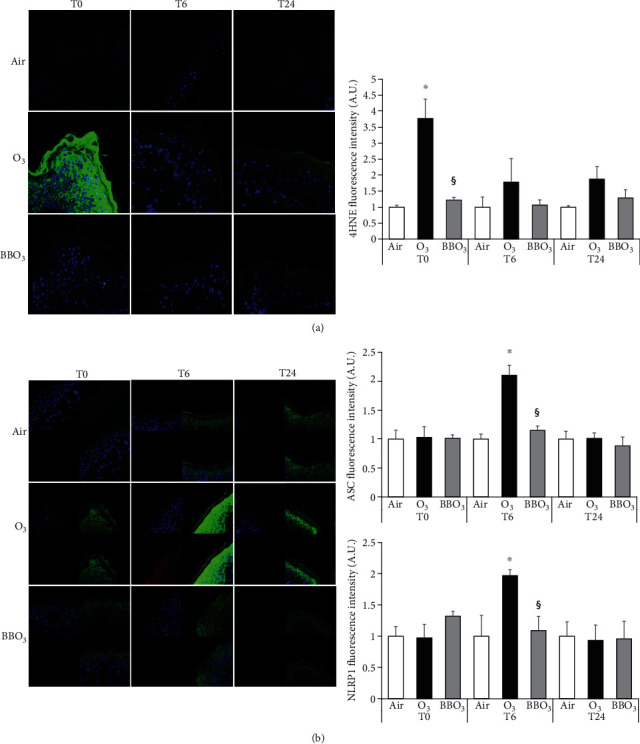
Blueberry (BB) extract prevents O_3_-induced inflammasome activation and oxidative stress in *ex vivo* human skin biopsies. (a) Immunofluorescence staining for 4HNE (green), DAPI (blue), and merge in *ex vivo* human skin biopsies exposed to O_3_ for 5 hours, 0.5 ppm, directly after exposure, 6 h and 24 h postexposure (40x magnification). Quantification of the fluorescence is depicted in the right panel. (b) Immunofluorescence staining of ASC (green), NLRP1 (red), DAPI (blue), and merge in *ex vivo* human skin explants pretreated with 100 *μ*g/ml of BB for 24 h then exposed to 0.5 ppm of O_3_ for 5 h at 0, 6, and 24 h postexposure (40x magnification). On the right panels, semiquantifications of the intensities of the signals of 4HNE (a) and ASC and NLRP1 (b) using ImageJ. Data are the results of three independent experiments. ^∗^*p* < 0.05 air vs. O_3_, ^§^*p* < 0.05 O_3_ vs. BBO_3_ by two-way ANOVA.

The biopsies were pretreated with 100 *μ*g/ml of BB extracts for 24 h and exposed to 0.5 ppm of O_3_ for 5 hours. As depicted in Figure 6(a); BB extract topical application was able to quench the increased protein levels of both ASC (green) and NLRP1 (red) and their nuclear colocalization 6 h after O_3_ exposure.

Since redox signaling is an important part of the inflammatory pathway [71], we also evaluated the level of 4 hydroxynonenal (4HNE) as a reliable marker of oxidative damage and lipid peroxidation. As depicted in Figure 6(b), O_3_ significantly increases the 4HNE levels immediately after the exposure and this effect was still visible at the later time points. BB extract pretreatment was able to prevent the 4HNE formation.

## 4. Discussion

It is now well accepted and documented that exposure to environmental pollution affects our health and this phenomenon is not localized to few urban centers, but it is a global emergency that has been estimated to decrease human life span by 10-15 years [72]. There are several pollutants to which living organisms are daily exposed, and among them, O_3_ has been shown to be one of the most toxic. It should be mentioned that the O_3_ derived from the stratospheric–tropospheric exchanges accounts for 20% of its tropospheric level. Nowadays, ozone is mainly produced by complex photochemical reactions involving solar radiation and anthropogenic pollutants [73, 74]. Photochemical ozone is formed by reactions involving solar radiation and anthropogenic pollutants (methane, nonmethane volatile organic compounds, and carbon monoxide) in the presence of nitrogen oxides while in less polluted environments, ozone is produced in the presence of sunlight (at wavelengths < 424 nm), through the photolysis of NO_2_ [73].

In addition, as recently reported, the levels of O_3_ are still increasing making the effect of this pollutant to our health a real concern not only for the present but even more for the future [75].

Being a gas, the toxicity of O_3_ has been well documented in the respiratory tract [76–78] and in the last 2 decades, its effect on cutaneous tissues has been also investigated since the skin is another organ directly exposed to this agent [2, 5–8].

O_3_ is a small molecule with strong oxidizing properties, with a redox potential of +2.07, able to oxidize a wide range of compounds including rubber. Indeed, it is too reactive to penetrate the tissues and as shown first for the lungs and more recently even for the skin [3], its ability to affect the tissues is mainly due to the generation of bioactive compounds that are formed by its interaction with the biological systems. Several studies have confirmed the ability of O_3_ to oxidize cell membrane, generating radical species that can damage the tissues. The generation of redox-mediated molecules in the stratum corneum can eventually affect the deeper layers of the skin, modulating important physiopathological skin pathways [12].

The cutaneous proinflammatory effect of O_3_ is mainly driven by its ability to activate nuclear factor kappa-light-chain enhancer (NF-*κ*B) which is a master regulator of proinflammatory responses. In the last few years, different players of inflammation have been studies and in particular, the inflammasome machinery has been recognized to play a key role in inflammatory skin conditions [25–27]. The activation of the inflammasome occurs in response to different stimuli including cell damage and pathogen-associated molecular patterns (DAMPs and PAMPS) as well as prooxidant stimuli such as O_3_ [17, 18], and also, in this case, NF-*κ*B activation plays an important role.

In our previous work, we have shown the inflammatory and oxidative effect of O_3_ on the skin and its ability to activate NF-*κ*B and increase oxidative damage [30]. The aim of the present study was to evaluate the eventual protective effect of BB extracts against O_3_-induced skin damage. Indeed, the exponential increase of pollution levels has aroused the need to find effective molecules that can be used as defensive agents against pollution-induced skin damage and premature cutaneous aging.

The antiaging research has been focused on an enormous range of products (natural or synthetic) with the aim to prevent, postpone, or reverse cutaneous aging signs. In general, those molecules can act in 2 ways, either quenching directly the radicals or by activating the cellular endogenous defensive system nuclear factor (erythroid-derived 2)-like 2 (NRF2) [79–81].

BB has been shown to be able to activate the nuclear factor erythroid-2-related factor 2 (NRF2) [82] and also quench radical formation [83] in several tissues, and it is now a general belief that BB beneficial effects are not limited to the “chemical” antioxidant properties, but mainly to its ability to induce an active cellular defense [84]. Therefore, as a follow-up of our recent study [30], in the present work, we were interested in evaluating the ability of BB extracts to prevent O_3_-induced skin inflammasome activation. In addition, BB topical application has been shown to stimulate collagen synthesis and prevent chronological skin aging [50–52, 85]. Among the wide array of natural antioxidant substances, we decided to focus our attention on blueberries because of their complex phytochemical profiles that have already shown to quench free radicals [42, 43]. The ability of BB to activate the NRF2 pathway could also indirectly affect the inflammasome activation as altered redox homeostasis can be also a trigger for this inflammatory pathway [18, 86]. We should also mention that the crosstalk between NRF2 and NF-*κ*B is crucial for maintaining the cellular responses and to resolve an inflammatory status. An imbalance between NRF2 and NF-*κ*B pathways can lead to chronic inflammation; therefore, the activation of NRF2 by BB extracts can prevent NF-*κ*B activation and modulate the tissue inflammatory status [87].

We were able to show that BB extracts were not toxic in the tested *in vitro* and *ex vivo* models. We found that BB extracts improved the recovery of scratch wound closure in O_3_-treated cells. The impairment of O_3_ on cutaneous wound healing has also been described previously by Lim et al., in aged mice [66], showing that the combination of aging and O_3_ exposure is able to reduce the levels of TGF*β*, a key player in tissue would healing [66, 88]. Considering that *in vitro* wound healing is mainly a test to evaluate the proliferative and migratory properties of the cells, we assessed whether O_3_ could affect any of those pathways and the eventual role of BB extracts. Our data showed that O_3_ was able to decrease both proliferation and migration of the cells while the BB pretreatment prevented these effects probably either via the activation of a cellular defensive system or less probably, by a direct interaction with the free radicals generated by O_3_. It is possible that the inhibition of proliferation is a consequence of their ability to activate NRF2 which has an inhibitory influence on NF-*κ*B that is one of the regulators of cyclin D1 expression, a key protein for cellular proliferation [61, 85, 89]. Although we did not evaluate the effect of BB on NFR2 activation, we showed a significant decrease in H_2_O_2_ by BB after O_3_ exposure, although the use of the probe DCF to measure H_2_O_2_ is controversial [90].

The overproduction of ROS and NF-*κ*B activation has often been linked to the development of inflammatory responses related to skin and other tissues. Recently, the involvement of the inflammasome machinery has been studied in relation of several skin conditions. The activation of the inflammasome requires two steps: a “priming” step that induces transcriptional upregulation of NLRP1, pro-IL-18, and pro-IL-1*β*, via NF-*κ*B and AP-1 signaling and posttranslational modifications of NLRP1 (phosphorylation, ubiquitination), followed by a second signal that yields to conformational changes in the NOD-like receptor structure which allows for the binding with the adaptor ASC and assembly of the whole complex [17].

To better understand the molecular mechanisms responsible for the BB effect on O_3_-induced inflammasome activation and oligomerization, we pretreated the keratinocytes with BB extracts, before exposing them to O_3_ and then, we evaluated transcript levels of IL-18, ASC, and Caspase 1 as well as ASC specks and oligomers. Our data evidenced the remarkable activity of BB in preventing the increased aforementioned mRNA levels and the oligomerization of the proteins NLRP1 and ASC, upon O_3_ exposure. These data confirmed the study by Wang et al. which demonstrated reduced gene expression levels of NLRP, Caspase 1, ASC, and proinflammatory cytokines IL-1*β*, TNF-*α*, IL-6, and iNOS in macrophages pretreated with BB extracts and then challenged with lipopolysaccharide [91].

As keratinocytes grown in monolayer culture do not undergo terminal differentiation, which results in the formation of the outermost layer of the skin, the stratum corneum (SC), and since this layer is the main target of O_3_ [92], we have performed our experiments also in RHE (reconstructed human epidermis). The use of RHE is strongly recommended to study *in vitro* cutaneous protection, since is not only represented by all the living epidermis layers but it also includes the SC [69], and our data confirmed the ability of BB extracts to prevent O_3_-induced Caspase 1 increase at both transcripts and protein levels. Although, RHE is a very reliable *in vitro* model, it still does not present all the SC layers making it more accessible to outdoor stressors. In addition, the lack of other skin cells such as melanocytes, immunity cells, might compromise skin responses [69].

For this reason, we further confirm our data in an *ex vivo* human skin explant which presents a normal skin barrier, functional basal layer, a mature SC, and all the cell types and cutaneous appendages present in in vivo human skin [93].

As demonstrated in 2D and 3D models, O_3_ was able to activate the inflammasome machinery also in the human skin explants, and BB extracts prevented this induction. In the specific, O_3_ exposure not only induced the oligomerization of the inflammasome components but also increased the levels of lipid peroxidation as detected by 4HNE protein adduct formation (as previously reported [16]) while the pretreatment with BB extract was able to prevent the O_3_-induced increase of 4HNE, ASC, and NLRP1 and their oligomerization. These data are in line not only with the theory that O_3_ is able to affect skin via the formation of lipid peroxidation products but also with the theory that this mechanism is redox mediated.

In conclusion, we have demonstrated, by the use of three different skin models, that BB extracts are able to prevent the inflammatory and oxidative skin damage induced by O_3_, making blueberries a possible innovative natural ingredient for new technologies against cutaneous pollution-induced damage.

## Data Availability

All data will be available upon request to the corresponding author.

## Supplementary Materials

Supplementary materials The cutaneous interaction with tropospheric O3 induced an increase in oxidative stress mediators (ROS) and a consequent induction of the inflammasome-related proteins NLRP1 and Caspase 1 that will lead to the assemble of the inflammasome scaffold and the activations of the inflammasome machinery. Blueberry extract (BB) treatment is able to prevent the inflammasome activation by O_3_.

## References

[1] World Health Organization (WHO) (2016).

[2] Fuks K. B., Woodby B., Valacchi G., Hautarzt D. (2019).

[3] Cross C. E., Valacchi G., Schock B. (2002). Environmental Oxidant Pollutant Effects on Biologic Systems. *American Journal of Respiratory and Critical Care Medicine*.

[4] Mustafa M. G. (1990). Biochemical basis of ozone toxicity. *Free Radical Biology and Medicine*.

[5] Valacchi G., Pagnin E., Corbacho A. M. (2004). In vivo ozone exposure induces antioxidant/stress-related responses in murine lung and skin. *Free Radical Biology and Medicine*.

[6] Valacchi G., Sticozzi C., Pecorelli A., Cervellati F., Cervellati C., Maioli E. (2012). Cutaneous responses to environmental stressors. *Annals of the New York Academy of Sciences*.

[7] Krutmann J., Bouloc A., Sore G., Bernard B. A., Passeron T. (2017). The skin aging exposome. *Journal of Dermatological Science*.

[8] Valacchi G., van der Vliet A., Schock B. C. (2002). Ozone exposure activates oxidative stress responses in murine skin. *Toxicology*.

[9] Lavigne E., Villeneuve P. J., Cakmak S. (2012). Air Pollution and Emergency Department Visits for Asthma in Windsor, Canada. *Canadian Journal of Public Health*.

[10] Kaplan G. G., Dixon E., Panaccione R. (2009). Effect of ambient air pollution on the incidence of appendicitis. *Canadian Medical Association Journal*.

[11] Lee S. H., Jeong S. K., Ahn S. K. (2006). An Update of the Defensive Barrier Function of Skin. *Yonsei Medical Journal*.

[12] Pecorelli A., Woodby B., Prieux R., Valacchi G. (2019). Involvement of 4‐hydroxy‐2‐nonenal in pollution‐induced skin damage. *BioFactors*.

[13] De Luca C., Valacchi G. (2010). Surface lipids as multifunctional mediators of skin responses to environmental stimuli. *Mediators of Inflammation*.

[14] Valacchi G., Muresan X. M., Sticozzi C. (2016). Ozone-induced damage in 3D-Skin Model is prevented by topical vitamin C and vitamin E compound mixtures application. *Journal of Dermatological Science*.

[15] Valacchi G., Sticozzi C., Belmonte G. (2015). Vitamin C Compound Mixtures Prevent Ozone-Induced Oxidative Damage in Human Keratinocytes as Initial Assessment of Pollution Protection. *PLOS ONE*.

[16] Valacchi G., Pecorelli A., Belmonte G. (2017). Protective Effects of Topical Vitamin C Compound Mixtures against Ozone- Induced Damage in Human Skin. *Journal of Investigative Dermatology*.

[17] Kelley N., Jeltema D., Duan Y., He Y. (2019). The NLRP3 Inflammasome: An Overview of Mechanisms of Activation and Regulation. *International Journal of Molecular Sciences*.

[18] Abais J. M., Xia M., Zhang Y., Boini K. M., Li P. L. (2015). Redox Regulation of NLRP3 Inflammasomes: ROS as Trigger or Effector?. *Antioxidants & Redox Signaling*.

[19] Freeman L. C., Ting J. P.-Y. (2016). The pathogenic role of the inflammasome in neurodegenerative diseases. *Journal of Neurochemistry*.

[20] Heneka M. T., Kummer M. P., Latz E. (2014). Innate immune activation in neurodegenerative disease. *Nature Reviews Immunology*.

[21] Heneka M. T. (2017). Inflammasome activation and innate immunity in Alzheimer's disease. *Brain Pathology*.

[22] Guo H., Callaway J. B., Ting J. P. Y. (2015). Inflammasomes: mechanism of action, role in disease, and therapeutics. *Nature Medicine*.

[23] Masters S. L., Latz E., O'Neill L. A. J. (2011). The Inflammasome in Atherosclerosis and Type 2 Diabetes. *Science Translational Medicine*.

[24] Duewell P., Kono H., Rayner K. J. (2010). NLRP3 inflammasomes are required for atherogenesis and activated by cholesterol crystals. *Nature*.

[25] Dombrowski Y., Peric M., Koglin S. (2011). Cytosolic DNA Triggers Inflammasome Activation in Keratinocytes in Psoriatic Lesions. *Science Translational Medicine*.

[26] de Sá D. C., Neto C. F. (2016). Inflammasomes and dermatology. *Anais Brasileiros de Dermatologia*.

[27] Beer H.-D., Contassot E., French L. E. (2014). The Inflammasomes in Autoinflammatory Diseases with Skin Involvement. *Journal of Investigative Dermatology*.

[28] Burian M., Yazdi A. S., Invest J. (2018). NLRP1 Is the Key Inflammasome in Primary Human Keratinocytes. *Journal of Investigative Dermatology*.

[29] Fenini G., Grossi S., Contassot E. (2018). Genome Editing of Human Primary Keratinocytes by CRISPR/Cas9 Reveals an Essential Role of the NLRP1 Inflammasome in UVB Sensing. *The Journal of Investigative Dermatology*.

[30] Ferrara F., Pambianchi E., Pecorelli A. (2020). Redox regulation of cutaneous inflammasome by ozone exposure. *Free Radical Biology and Medicine*.

[31] Birben E., Sahiner U. M., Sackesen C., Erzurum S., Kalayci O. (2012). Oxidative Stress and Antioxidant Defense. *World Allergy Organization Journal*.

[32] Circu M. L., Aw T. Y. (2010). Reactive oxygen species, cellular redox systems, and apoptosis. *Free Radical Biology and Medicine*.

[33] Hanschmann E. M., Godoy J. R., Berndt C., Hudemann C., Lillig C. H. (2013). Thioredoxins, Glutaredoxins, and Peroxiredoxins—Molecular Mechanisms and Health Significance: from Cofactors to Antioxidants to Redox Signaling. *Antioxidants & Redox Signaling*.

[34] Poljsak B., Šuput D., Milisav I. (2013). Achieving the Balance between ROS and Antioxidants: When to Use the Synthetic Antioxidants. *Oxidative Medicine and Cellular Longevity*.

[35] Krol E. S., Kramer-Stickland K. A., Liebler D. C. (2000). Photoprotective Actions of Topically Applied Vitamin E∗. *Drug Metabolism Reviews*.

[36] Burke K. E., Clive J., Combs G. F., Commisso J., Keen C. L., Nakamura R. M. (2000). Effects of Topical and Oral Vitamin E on Pigmentation and Skin Cancer Induced by Ultraviolet Irradiation in Skh:2 Hairless Mice. *Nutrition and Cancer*.

[37] Działo M., Mierziak J., Korzun U., Preisner M., Szopa J., Kulma A. (2016). The Potential of Plant Phenolics in Prevention and Therapy of Skin Disorders. *International Journal of Molecular Sciences*.

[38] Souyoul S. A., Saussy K. P., Lupo M. P. (2018). Nutraceuticals: A Review. *Dermatology and Therapy*.

[39] Lin J.-Y., Selim M. A., Shea C. R. (2003). UV photoprotection by combination topical antioxidants vitamin C and vitamin E. *Journal of the American Academy of Dermatology*.

[40] Thiele J. J., Ekanayake-Mudiyanselage S. (2007). Vitamin E in human skin: Organ-specific physiology and considerations for its use in dermatology. *Molecular Aspects of Medicine*.

[41] Mistry N. (2017). Guidelines for Formulating Anti-Pollution Products. *Cosmetics*.

[42] Kalt W., Cassidy A., Howard L. R. (2019). Recent Research on the Health Benefits of Blueberries and Their Anthocyanins. *Advances in Nutrition*.

[43] Tsuda T. (2012). Dietary anthocyanin-rich plants: biochemical basis and recent progress in health benefits studies. *Molecular Nutrition & Food Research*.

[44] Lee S., Keirsey K. I., Kirkland R., Grunewald Z. I., Fischer J. G., de la Serre C. B. (2018). Blueberry Supplementation Influences the Gut Microbiota, Inflammation, and Insulin Resistance in High-Fat-Diet–Fed Rats. *The Journal of Nutrition*.

[45] Liu Y., Tikunov Y., Schouten R. E., Marcelis L. F. M., Visser R. G. F., Bovy A. (2018). Anthocyanin Biosynthesis and Degradation Mechanisms in Solanaceous Vegetables: A Review. *Frontiers in Chemistry*.

[46] Wolfe K. L., Kang X., He X., Dong M., Zhang Q., Liu R. H. (2008). Cellular Antioxidant Activity of Common Fruits. *Journal of Agricultural and Food Chemistry*.

[47] Stevenson D., Scalzo J. (2012). Anthocyanin composition and content of blueberries from around the world. *Journal of Berry Research*.

[48] Kalt W., Ryan D. A. J., Duy J. C., Prior R. L., Ehlenfeldt M. K., Vander Kloet S. P. (2001). Interspecific Variation in Anthocyanins, Phenolics, and Antioxidant Capacity among Genotypes of Highbush and Lowbush Blueberries (VacciniumSectioncyanococcusspp.). *Journal of Agricultural and Food Chemistry*.

[49] Grace M. H., Ribnicky D. M., Kuhn P. (2009). Hypoglycemic activity of a novel anthocyanin-rich formulation from lowbush blueberry, _Vaccinium angustifolium_ Aiton. *Phytomedicine*.

[50] Grether-Beck S., Krutmann J., Wilkens K., D'Amato K. (2017). Effect of a Blueberry-Derived Antioxidant Matrix on Infrared-A Induced Gene Expression in Human Dermal Fibroblasts. *Journal of Drugs in Dermatology*.

[51] Lau F. C., Bagchi M., Zafra-Stone S., Bagchi D. (2009). The Benefits of Antioxidant-Rich Fruits on Skin Health. *Nutritional Cosmetics*.

[52] Bagchi M., Zafra-Stone S., Losso J. N. (2007). Anti-angiogenic functional and medicinal foods. *Anti-angiogenic functional and medicinal foods*.

[53] Lila M. A., Lila K. D., Quideau E. S., Freitas V., Reed J. (2020). *Recent Advances in Polyphenol ResearchVolume 7, Chapter 3*.

[54] Xu F., Yan S., Wu M. (2011). Ambient ozone pollution as a risk factor for skin disorders. *British Journal of Dermatology*.

[55] Kousha T., Valacchi G., Toxicol J. (2015). The air quality health index and emergency department visits for urticaria in Windsor, Canada. *Journal of Toxicology and Environmental Health*.

[56] Benedusi M., Frigato E., Beltramello M., Bertolucci C., Valacchi G. (2018). Circadian clock as possible protective mechanism to pollution induced keratinocytes damage. *Mechanisms of Ageing and Development*.

[57] Grace M. H., Warlick C. W., Neff S. A., Lila M. A. (2014). Efficient preparative isolation and identification of walnut bioactive components using high-speed counter-current chromatography and LC-ESI-IT-TOF- MS. *Food Chemistry*.

[58] Grace M. H., Esposito D., Dunlap K. L., Lila M. A. (2014). Comparative Analysis of Phenolic Content and Profile, Antioxidant Capacity, and Anti-inflammatory Bioactivity in Wild Alaskan and Commercial Vaccinium Berries. *Journal of Agricultural and Food Chemistry*.

[59] Magnani N. D., Muresan X. M., Belmonte G. (2016). Skin Damage Mechanisms Related to Airborne Particulate Matter Exposure. *Toxicological Sciences*.

[60] Cervellati F., Muresan X. M., Sticozzi C. (2014). Comparative effects between electronic and cigarette smoke in human keratinocytes and epithelial lung cells. *Toxicology in Vitro*.

[61] Valacchi G., Pecorelli A., Mencarelli M. (2009). Rottlerin: a multifaced regulator of keratinocyte cell cycle. *Experimental Dermatology*.

[62] Maria M. X., Claudia S., Giuseppe B. (2018). *Archives of Biochemistry and Biophysics*.

[63] Valacchi G., Sticozzi C., Zanardi I. (2016). Ozone mediators effect on “in vitro” scratch wound closure. *Free Radical Research*.

[64] Sticozzi C., Pecorelli A., Romani A. (2018). Tropospheric ozone affects SRB1 levels via oxidative post-translational modifications in lung cells. *Free Radical Biology and Medicine*.

[65] Muresan X. M., Sticozzi C., Belmonte G., Savelli V., Evelson P., Valacchi G. (2018). Modulation of cutaneous scavenger receptor B1 levels by exogenous stressors impairs "in vitro" wound closure. *Mechanisms of Ageing and Development*.

[66] Lim Y., Phung A. D., Corbacho A. M. (2006). Modulation of cutaneous wound healing by ozone: differences between young and aged mice. *Toxicology letters*.

[67] Lebwohl M. (2018). Psoriasis. *Annals of Internal Medicine*.

[68] Haass N. K., Smalley K. S. M., Li L., Herlyn M. (2005). Adhesion, migration and communication in melanocytes and melanoma. *Pigment Cell Research*.

[69] Rossi A., Appelt-Menzel A., Kurdyn S., Walles H., Groeber F. (2015). Generation of a Three-dimensional Full Thickness Skin Equivalent and Automated Wounding. *Journal of Visualized Experiments*.

[70] Dick M. S., Sborgi L., Rühl S., Hiller S., Broz P. (2016). ASC filament formation serves as a signal amplification mechanism for inflammasomes. *Nature Communications*.

[71] Valacchi G., Virgili F., Cervellati C., Pecorelli A. (2018). OxInflammation: From Subclinical Condition to Pathological Biomarker. *Frontiers in Physiology*.

[72] Lelieveld J., Pozzer A., Pöschl U., Fnais M., Haines A., Münzel T. (2020). Inappropriate evaluation of methodology and biases by P. Morfeld and T.C. Erren.. *Cardiovascular Research*.

[73] Alvim-Ferraz M. C. M., Sousa S. I. V., Pereira M. C., Martins F. G. (2006). Contribution of anthropogenic pollutants to the increase of tropospheric ozone levels in the Oporto Metropolitan Area, Portugal since the 19th century. *Environmental Pollution*.

[74] Marenco A., Gouget H., Nedelec P., Pages J. P., Karcher F. (1994). Evidence of a long-term increase in tropospheric ozone from Pic du Midi data series: Consequences: Positive radiative forcing. *Journal of Geophysical Research*.

[75] Lin M., Horowitz L. W., Xie Y. (2020). Vegetation feedbacks during drought exacerbate ozone air pollution extremes in Europe. *Nature Climate Change*.

[76] Kelly F. J., Mudway I. S. (2003). Protein oxidation at the air-lung interface. *Amino Acids*.

[77] Kazemiparkouhi F., Eum K.-D., Wang B., Manjourides J., Suh H. H. (2020). Long-term ozone exposures and cause-specific mortality in a US Medicare cohort. *Journal of Exposure Science and Environmental Epidemiology*.

[78] Luong L. M. T., Phung D., Dang T. N., Sly P. D., Morawska L., Thai P. K. (2018). Seasonal association between ambient ozone and hospital admission for respiratory diseases in Hanoi, Vietnam. *PLoS One*.

[79] Si H., Liu D. (2014). Dietary antiaging phytochemicals and mechanisms associated with prolonged survival. *Journal of Nutritional Biochemistry*.

[80] BLACK H. O. M. E. R. S., MATHEWS-ROTH M. I. C. H. E. L. I. N. E. M. (1991). PROTECTIVE ROLE OF BUTYLATED HYDROXYTOLUENE AND CERTAIN CAROTENOIDS IN PHOTOCARCINOGENESIS. *Photochemistry and Photobiology*.

[81] de la Vega M. R., Krajisnik A., Zhang D., Wondrak G. (2017). Targeting NRF2 for Improved Skin Barrier Function and Photoprotection: Focus on the Achiote-Derived Apocarotenoid Bixin. *Nutrients*.

[82] Tang J. S., Vissers M. C. M., Anderson R. F. (2018). Bioavailable Blueberry-Derived Phenolic Acids at Physiological Concentrations Enhance Nrf2-Regulated Antioxidant Responses in Human Vascular Endothelial Cells. *Molecular Nutrition & Food Research*.

[83] Dróżdż P., Šėžienė V., Pyrzynska K. (2017). Phytochemical Properties and Antioxidant Activities of Extracts from Wild Blueberries and Lingonberries. *Plant Foods for Human Nutrition*.

[84] Song Y., Huang L., Yu J. (2016). Effects of blueberry anthocyanins on retinal oxidative stress and inflammation in diabetes through Nrf2/HO-1 signaling. *Journal of Neuroimmunology*.

[85] Shin S., Cho S. H., Park D., Jung E. (2020). Anti‐skin aging properties of protocatechuic acid in vitro and in vivo. *Journal of Cosmetic Dermatology*.

[86] Rubartelli A., Gattorno M., Netea M. G., Dinarello C. A. (2011). Interplay between redox status and inflammasome activation. *Trends in Immunology*.

[87] Wardyn J. D., Ponsford A. H., Sanderson C. M. (2015). Dissecting molecular cross-talk between Nrf2 and NF-*κ*B response pathways. *Biochemical Society Transactions*.

[88] Lu L., Chen S. S., Zhang J. Q., Ramires F. J., Sun Y. (2004). Activation of nuclear factor-*κ*B and its proinflammatory mediator cascade in the infarcted rat heart. *Biochemical and Biophysical Research Communications*.

[89] Huang W. Y., Liu Y. M., Wang J., Wang X. N., Li C. Y. (2014). Anti-Inflammatory Effect of the Blueberry Anthocyanins Malvidin-3-Glucoside and Malvidin-3-Galactoside in Endothelial Cells. *Molecules*.

[90] Forman H. J., Augusto O., Brigelius-Flohe R. (2015). Even free radicals should follow some rules: a guide to free radical research terminology and methodology. *Free Radical Biology and Medicine*.

[91] Wang H., Guo X., Liu J., Li T., Fu X., Liu R. H. (2017). Comparative suppression of NLRP3 inflammasome activation with LPS-induced inflammation by blueberry extracts (Vaccinium spp.). *RSC Advances*.

[92] Packer L., Valacchi G. (2002). Antioxidants and the Response of Skin to Oxidative Stress: Vitamin E as a Key Indicator. *Skin Pharmacology and Physiology*.

[93] Sidgwick G. P., McGeorge D., Bayat A. (2016). Functional testing of topical skin formulations using an optimised ex vivo skin organ culture model. *Archives of Dermatological Research*.

