# Supplementary material for: Blueberry Extracts as a Novel Approach to Prevent Ozone-Induced Cutaneous Inflammasome Activation
Source: Oxid Med Cell Longev. 2020 Aug 13;2020:9571490. doi: 10.1155/2020/9571490 (PMC7443250; doi:10.1155/2020/9571490)
Supplement: Supplementary materials — The cutaneous interaction with tropospheric O3 induced an increase in oxidative stress mediators (ROS) and a consequent induction of the inflammasome-related proteins NLRP1 and Caspase 1 that will lead to the assemble of the inflammasome scaffold and the activations of the inflammasome machinery. Blueberry extract (BB) treatment is able to prevent the inflammasome activation by O3. [file 9571490.f1.docx]

**GRAPHICAL ABSTRACT**


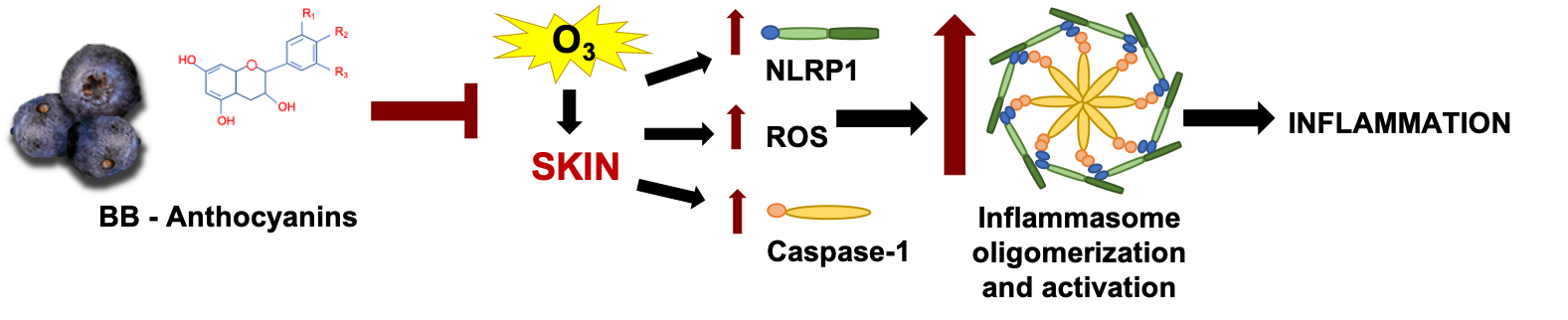


The cutaneous interaction with tropospheric O_3_ induced an increase in oxidative stress mediators (ROS) and a consequently induction of the inflammasome related proteins NLRP1 and Caspase 1 that will lead to the assemble of the inflammasome scaffold and the activations of the inflammasome machinery. Blueberries extracts (BB) treatment is able to prevent the inflammasome activation by O3.
